# A Pro-Atherogenic HDL Profile in Coronary Heart Disease Patients: An iTRAQ Labelling-Based Proteomic Approach

**DOI:** 10.1371/journal.pone.0098368

**Published:** 2014-05-23

**Authors:** Li-rong Yan, Dong-xue Wang, Hong Liu, Xiao-xing Zhang, Hui Zhao, Lu Hua, Ping Xu, Yi-shi Li

**Affiliations:** 1 Key Laboratory of Clinical Trial Research in Cardiovascular Drugs, Ministry of Health, State Key Laboratory of Cardiovascular Diseases, FuWai Hospital, National Center for Cardiovascular Diseases, Chinese Academy of Medical Sciences and Peking Union Medical College, Beijing, China; 2 Department of Cardiology, Wuxi People' Hospital Affiliated to Nanjing Medical University, Wuxi, Jiangsu, China; University of Louisville, United States of America

## Abstract

**Objectives:**

This study aims to compare the protein composition of high-density lipoprotein (HDL) particles in coronary heart disease (CHD) patients and controls by proteomic methods.

**Background:**

HDL has been reported to exert pro-atherogenic properties in CHD patients. Accumulating evidence indicates that HDL composition, rather than the HDL-C level, determines its functions. The changes in HDL composition involved in the conversion of anti-atherogenic to pro-atherogenic properties in CHD patients are currently unknown.

**Methods and Results:**

iTRAQ combined with nanoLC-MS/MS was performed to obtain a differential expression profile of the HDL pooled samples of the male age-matched CHD patients and controls (n = 10/group). Of the 196 proteins identified in the examined HDL, 12 were differentially expressed between the CHD patients and the controls, including five up-regulated proteins and seven down-regulated proteins. Using GO analysis, we determined that the up-regulated proteins were mostly involved in inflammatory reactions, displaying a potential pro-atherogenic profile. In contrast, the down-regulated proteins were mostly involved in lipid metabolism processes, displaying anti-atherogenic properties. To confirm the proteomic results, serum amyloid A (SAA) and apoC-I were selected and quantified by ELISA, in the same population as the proteomic analysis, as well as another independent population (n = 120/group). Consistent with the proteomic results, the amount of SAA was significantly increased, and apoC-I was significantly decreased in the HDL particles of CHD patients compared with those of controls (*P*<0.05).

**Conclusions:**

Our study shows that the HDL proteome changes to a pro-atherogenic profile in CHD patients, which might compromise the protective effects of HDL. Proteomic analysis of HDL composition may provide more relevant information regarding their functional properties than steady-state HDL-C levels.

## Introduction

Coronary heart disease (CHD) is one of the leading causes of morbidity and mortality in China, as well as in the rest of the world, and early detection and diagnosis are the most effective measures for preventing the progression of the disease [1]. Lipids and lipoproteins from plasma have been powerful risk factors for predicting CHD [2], [3]. One of them, high-density lipoprotein cholesterol (HDL-C), is a complex heterogeneous mixture of particles consisting of lipids and smaller proteins [4]. Substantial epidemiological studies have demonstrated that HDL-C level is a strong, independent, and inverse risk factor for CHD [5]–[7]. This is mainly because of the effects of HDL on preventing atherosclerosis by promoting cholesterol efflux, reducing oxidation, attenuating vascular inflammation, and improving vascular endothelial function [8]. However, there is accumulating evidence that HDL composition determines its functional properties, rather than the circulating HDL-C level [4]. ApoA-I in HDL has been identified as being responsible for reverse cholesterol transport and reverse atherosclerosis, and paraoxonase 1 in HDL has been found to have anti-oxidant properties [9]. In addition, studies have indicated that elevated amounts of serum amyloid A (SAA) in HDL render it pro-atherogenic [10], [11]. It has also been demonstrated that overexpression of apoA-II in HDL fractions advanced atherosclerosis even though the plasma levels of HDL-C were elevated [12].

Inflammation plays a major role in changing the composition and function of circulating HDL [13]. Indeed, it is well accepted that there is a significant link between CHD and inflammation [14], [15]. Several recent studies have provided evidence that HDL exerts pro-inflammatory properties and loses its protective functions in the context of CHD [16], [17]. These findings are supported by the studies indicating that inflammation generates dysfunctional or even pro-atherogenic forms of HDL by promoting phospholipid depletion and enrichment with pro-inflammatory proteins such as SAA or complement component 3 [18], [19]. The composition changes promoting the conversion of anti-inflammatory HDL to pro-inflammatory HDL in CHD have not been fully identified. Knowing the molecular profiles that distinguish pro-inflammatory and anti-inflammatory HDL may facilitate a better understanding of the alterations in HDL's protein cargo, which adversely affect HDL normal anti-atherosclerosis functions.

To test the hypothesis that HDL particles are remodeled under inflammatory conditions in CHD without lipid regulating drugs, we compared the protein composition of HDL particles isolated from CHD patients with those isolated from controls, using the isobaric tag for relative and absolute quantification (iTRAQ) technique combined with nanoflow liquid chromatography-tandem mass spectrometry (nanoLC-MS/MS). This is one of the most highly sensitive proteomic technologies, as it can detect and quantitatively analyze low abundance proteins in complex biological samples [20]–[22]

## Methods

### Study subjects and Experimental strategy

A total of 260 subjects were enrolled in this study, including 130 CHD patients and 130 healthy controls. All of the patients and controls were recruited from patients referred to the FuWai Hospital, Beijing, China, between July 2011 and June 2012. The diagnosis of CHD was defined according to WHO criteria as follows: significant stenosis ≥50% in at least one major coronary artery determined by percutaneous coronary angiography. Patients with acute myocardial infarction were excluded. Control subjects were determined to be free of CHD by percutaneous coronary angiography or computed tomographic angiography. Exclusion criteria included uncontrolled hypertension, triglycerides ≥5.0 mmol/L, severe obesity (BMI≥30), presence of thyroid, hepatic or renal disease, any chronic or acute infections, inflammatory illness, autoimmune disease and any type of cancers. In addition, subjects were excluded if they had regularly received lower lipid therapy in the previous two months, such as statins. All subjects were free of anti-inflammatory medications. The study protocol was approved by the Ethics Committee of the FuWai Hospital (Approval No. 2012-382) and conducted according to the principles expressed in the Declaration of Helsinki. Written informed consents were obtained from all subjects before enrollment. This trial has been registered at http://clinicaltrials.gov/ (Identification Number: NCT01543308).

In the initial discovery stage, iTRAQ combined with nanoLC-MS/MS was performed to obtain a global and a differential expression profile of the HDL pooled samples between the two groups in cohort 1, which included 10 male patients with CHD and 10 age- and sex-matched controls. Then, in the validation stage, target proteins were selected on the basis of the proteome maps and quantified by enzyme-linked immunosorbent assay (ELISA), in cohort 1 and an independent cohort 2 of 120 CHD patients and 120 controls. Blood samples were collected after overnight fasting and prior to percutaneous coronary angiography.

### Lipid analysis

Cholesterol, triglyceride, LDL-C and HDL-C concentrations were determined enzymatically using an autoanalyzer (Cobas c 311, Roche, Germany). HDL-C concentrations were determined after precipitation of apoB-containing lipoproteins by dextran sulfate with polyethylene glycol. LDL-C concentrations were calculated by the Friedewald equation [23]. ApoB and high-sensitivity C-reactive protein (hs-CRP) concentrations were determined by nephelometry (Beckman Immage Analyzer, USA).

### Isolation of plasma HDL by ultracentrifugation and identification of HDL-purity

**Figure 1:**
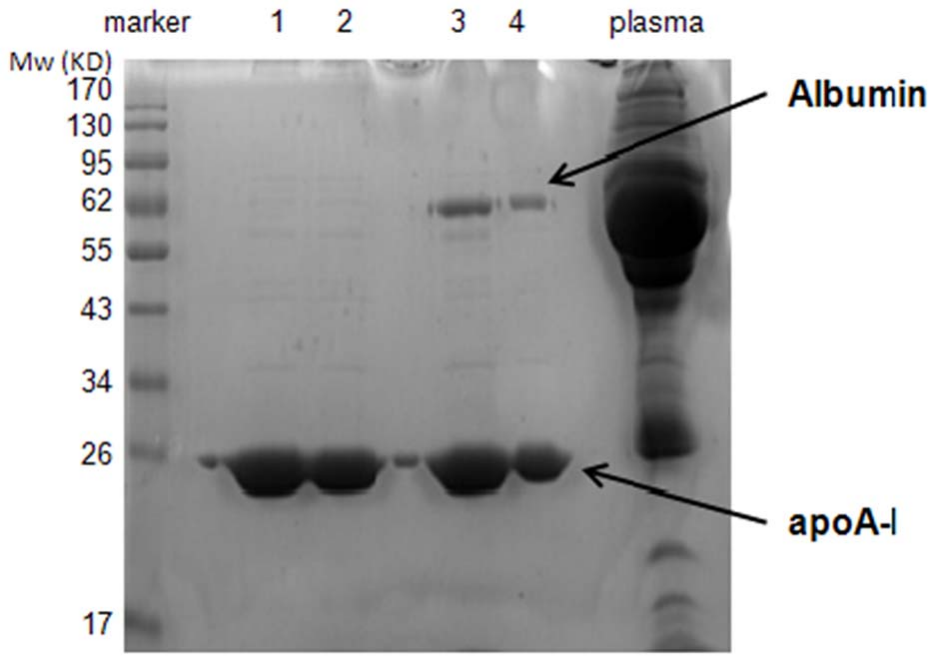
Analysis of HDL samples by SDS-PAGE (12%). Proteins were detected by staining with Coomassie blue (Invitrogen, the Netherlands). Lane 1 and 2 are representative of 25 µg high-abundance protein depleted HDL sample pools (from CHD group and control group for iTRAQ analysis), confirming the efficiency of high-abundance protein depletion. Lane 3 and 4 indicate two pooled HDL samples (each pooled with 10 random samples), confirming the purity of HDL isolated by ultracentrifugation; Lane of plasma, 25 µg protein of plasma is loaded as control.

Regarding the density cutoff of ultracentrifugation technique for HDL isolation, 1.063<d<1.24 g/ml and 1.063<d<1.21 g/ml are the two common reported cutoff values [24]–[28]. Using our lab instrumentation (80 Tirotor, Optima TM LE-80K ultracentrifuge, Beckman Coulter, USA), we tested both density cutoffs, and found that the amount of HDL-associated apoA-I was much less by using 1.063<d<1.21 g/ml compared to 1.063<d<1.24 g/ml, so we used the density cutoff of 1.063<d<1.24 g/ml (Table S1). HDL was isolated from 1.5 ml fresh EDTA-plasma of all the participants (n = 260) by sequential ultracentrifugation using potassium bromide (KBr) [24]. Briefly, a density gradient (the upper was 1.063 g/ml KBr solution and the lower was 1.24 g/ml plasma adjusted with KBr) was made in centrifuge tubes, and then the samples were centrifuged at 65,000 rpm for 5 h at 10°C. VLDL and LDL were sequentially removed by adjusting the supernatant density to 1.063 g/ml; and HDL fractions were obtained by adjusting the infranatant to 1.24 g/ml. HDL fractions were desalted, dried by vacuum centrifugation and stored at −80°C. The purity of the HDL fraction was assessed by 1-dimensional electrophoresis (1-DE) in two pooled samples (each pooled with 10 random samples). The protein concentration in isolated HDL samples was quantified in duplicate using a BCA protein assay kit (Applygen Technologies Inc, China). Then, 25 µg HDL sample were separated by sodium dodecyl sulfate polyacrylamide gel electrophoresis (SDS-PAGE) on a 12% gel (Figure 1). In addition, nephelometric analysis was also used to identify the purity of 20 random HDL samples using an Immage Nephelometric Analyzer (Beckman Coulter) according to the instructions. All HDL samples exhibited an apoA-I level from 1.02–1.94 g/L and an apolipoprotein B level under the limit of detection of <0.3 g/L.(Table S2).

### HDL fraction preparation for iTRAQ analysis

#### High-abundance protein depletion

To enhance the detection and identification of medium- and low-abundance proteins for iTRAQ analysis, high-abundance proteins, such as albumin and IgG, were removed from the HDL samples using a ProteoExtrct Albumin/IgG Removal kit (Calbiochem, Merck KGaA, Germany). The protein concentrations in high-abundance depleted HDL samples were determined using a bicinchoninic acid kit.

#### Sample Pooling

Sample pooling has often been used in proteomics experiments, as it serves to reduce overall variability by minimizing individual heterogeneity [20], [27], [29]. An equal amount of 10 HDL samples from both the control and CHD groups were combined to generate two sample pools. SDS-PAGE was used to evaluate the efficiency of high-abundance protein depletion in the two pooled samples (Figure 1).

### iTRAQ Labeling

Protein digestion and iTRAQ labeling were performed according to the manufacturer's protocol (iTRAQ Multi-plex kit, Applied Biosystems, USA). Briefly, the proteins of the HDL pools from the two groups were precipitated using acetone, and then dissolved into 20 µl of dissolution buffer (AB Sciex USA). After dissolution, the protein content of each sample pool was quantified via Bradford assay. Eighty micrograms of the processed HDL were taken from each sample solution and digested with trypsin with a ratio of protein∶trypsin  =  30∶1 at 37°C for 16 hours. After trypsin digestion, peptides from the control and the CHD groups were labeled with iTRAQ tags 114 and 116 for 1 hour, respectively. The labeled peptide pools were then mixed and dried by vacuum centrifugation.

### Separation by high pressure liquid chromatography (HPLC)

HPLC was performed with a RIGOL L3220 HPLC pump system. The iTRAQ-labeled peptide mixtures were reconstituted with 4 ml of buffer A (2% acetonitrile[ACN], 98%H_2_O, pH 10.0) and loaded onto a 4.6×250 mm Agela Venusil XBP C18 column containing 5-µm particles. The peptides were eluted at a flow rate of 0.7 ml/min with a gradient of 5–8% buffer B (98% ACN, 2%H_2_O, pH 10.0) for 1 min, 8–32% buffer B for 24 min, 32–95% buffer B for 2 min, 95% buffer B for 4 min, and 95–5% buffer B for 1 min. Elution was monitored by measuring the absorbance at 214 nm, and fractions were collected every minute. The eluted peptides were pooled into 8 fractions, and then vacuum-dried.

### LC -MS/MS Analysis

LC−MS/MS analysis was conducted with a Q-Exactive quadrupole-Orbitrap mass spectrometer (Thermo Fisher Scientific, USA) coupled with an EASY-nanoLC-1000 system. The mobile phases consisted of solvent A (98% water, 1.9% ACN, and 0.1% formic acid) and solvent B (98% ACN, 1.9% water, and 0.1% formic acid). The HPLC fractionated peptides were dissolved in solvent A and loaded on a C18 trap column (100 µm×20 mm×5 µm). Chromatographic separation was performed with a C18 column (75 µm×150 mm×3 µm). The gradient was delivered at 450 nl/min and consisted of a linear gradient of mobile phase B initiating from solvent B, 3–90% over 78 min. The mass spectrometer was operated in positive ion mode with a resolution of 70 000 using a source temperature of 320°C. The applied electrospray voltage was 1.9 kV. For MS scans, the m/z scan range was 300 to 1400 Da.

### Database Search and Bioinformatics

The MS/MS obtained spectra were searched against the NCBI reference sequence (RefSeq) database (version 20110124, Human) using Proteome Discoverer software (Thermo Fisher Scientific, USA, version 1.3) with the MASCOT (Matrix Science, London, U.K.; version 2.1) search algorithm. The search parameters for tryptic cleavage and accuracy are built-in functions of the software. For protein identification and quantification, a peptide mass tolerance of 15 ppm was allowed for intact peptide masses and 20 mmu for fragment ions. Two missed cleavages were allowed in the trypsin digests. Carbamidomethylation of cysteine was considered as a fixed modification, and the conversions of N-terminal glutamine to pyroglutamic acid and methionine oxidation were considered variable modifications.

All identified peptides had an ion score above the Mascot peptide identity threshold (a high confidence score of 99% and a low false discovery rate (FDR) of 1%), and a target protein was considered identified if at least two such unique peptide matches were apparent for the protein. For protein-abundance ratios measured using iTRAQ (116/114), we set a 1.5-fold up-regulation and 0.75-fold down-regulation change as the threshold and a two-tailed p-value <0.05 to identify significant changes. Gene Ontology (GO) functional classifications were analyzed with DAVID software (http://david.abcc.ncifcrf.gov) and GO enrichment analysis was performed to identify GO terms that were significantly enriched in differentially expressed proteins.

### Confirmation of MS/MS results by ELISA

To establish the clinical application of our findings, apolipoprotein C-I (apoC-I) and serum amyloid A2 (SAA2) expressions in HDL were examined by ELISA. SAA2 is a precursor and further processed into the mature and active form of SAA [30]. Since there is no commercial biological reagent for SSA2, SAA was measured. Commercial ELISA kits (AssayPro LLC, Saint Charles, USA; and Invitrogen Life Technologies, Carlsbad, USA) were used to quantify apoC-I and SAA according to the manufacturer's instructions, respectively. All measurements were performed in duplicate.

### Statistical analysis

All statistics were performed using SPSS version 18.0 (SPSS Inc, Chicago, IL). The descriptive data are presented as the mean ± SD, and the continuous and nominal variables are presented as frequencies respectively. The continuous data were compared using the Student's *t*-test or Mann-Whitney test, and the nominal data were compared using the Chi-square test. A multiple linear regression analysis was performed to determine the association between the expression of SAA and apoC-I in HDL fractions (as a dependent variable respectively), and the difference variables of plasma hs-CRP and a history of smoking between the two groups. A *P* value <0.05 was considered significant.

## Results

### Proteomic HDL analysis of the discovery population

The clinical characteristics of the study subjects in the discovery stage are displayed in Table 1. Control and CHD subjects were matched for gender, age, BMI and lipid profiles, including HDL-C and apoA-I. Patients with CHD showed elevated levels of high sensitivity C-reactive protein (hs-CRP) relative to controls (*P* = 0.013). All subjects in the two groups were non-diabetic. There were no statistical differences regarding medical treatment of antihypertensive agents or aspirin between the two groups.

**Table 1:** Clinical characteristics of study population.

	Discovery cohort			Validation cohort		
	Controls (n = 10)	CHD (n = 10)	*P*-value	Controls (n = 120)	CHD (n = 120)	*P*-value
Gender (male)	10 (100%)	10 (100%)	NS	80 (66.7%)	89 (74.2%)	NS
Age (years)	56.4±5.2	57.7±4.5	NS	52.8±8.5	54.6±9.8	NS
BMI (kg/m^2^)	25.5±2.5	26.0±3.3	NS	25.7±3.1	25.8±3.2	NS
Plasma						
Cholesterol (mmol/L)	4.1±0.7	4.0±0.9	NS	4.4±0.7	4.3±1.0	NS
Triglyceride (mmol/L)	1.2±0.4	1.4±0.1	NS	1.6±0.9	1.8±1.0	NS
HDL-C (mmol/L)	1.1±0.2	1.0±0.2	NS	1.2±0.3	1.0±0.2	<0.001^a^
LDL-C (mmol/L)	2.5±0.6	2.4±0.8	NS	2.8±0.8	2.7±0.9	NS
ApoA-I (mmol/L)	1.4±0.1	1.4±0.2	NS	1.5±0.3	1.4±0.2	NS
ApoB (mmol/L)	1.0±0.2	0.9±0.3	NS	1.0±0.2	1.1±0.3	NS
hs-CRP (mg/L)	1.4±1.9	4.1±4.2	0.013	2.1±2.5	3.9±4.1	<0.001^a^
Smokers	2 (20%)	2 (20%)	NS	41 (34.2%)	75 (62.5%)	<0.001^a^
Diabetes	0 (0%)	0 (0%)	NS	15 (12.5%)	16 (13.3%)	NS
Hypertension	4 (40%)	5 (50%)	NS	48 (47.1%)	71 (59.2%)	NS
Antidiabetic agents	0 (0%)	0 (0%)	NS	15 (12.5%)	16 (13.3%)	NS
Antihypertensive agents	4 (40%)	5 (50%)	NS	48 (47.1%)	71 (59.2%)	NS
Aspirin	4 (40%)	6 (60%)	NS	70 (60.0%)	87 (72.5%)	NS

Data presented as the number (%) or mean ± SD;

a *P*<0.05, NS denotes not significant between CHD patients and controls.

Using iTRAQ and 2D LC−MS/MS, we compared the two-pooled HDL samples from the CHD patients and the controls. To be identified as true-positive, proteins were required to contain two or more peptides with a 99% confidence score and a 1% local FDR in each of the two analyzed groups. A total of 196 high-confidence proteins, corresponding with the 1101 unique peptides, were identified in the HDL fractions isolated by ultracentrifugation. (Table S3)

Of those identified, the majority of HDL-associated proteins were common between control and CHD subjects, and only 12 proteins in the CHD patients displayed a differential expression with iTRAQ reporter ion intensities >1.5- or <0.75-fold (with a *P*-value ≤0.05) relative to the control samples. Among them, 5 proteins were up-regulated, and 7 were down-regulated (Table 2).

**Table 2:** Differentially expressed proteins in CHD patients identified by iTRAQ.

GI number	Protein names	Gene symbol	Coverage (%)	Unique peptides	Peptides Spectrum match	iTRAQ ratio of CHD/control	*P*-value
**Up-regulated**							
156071459	ADP/ATP translocase 2	ANT 2	8.72	2	3	1.628	0.006
4885371	histone H1	H1F0	4.12	2	3	1.596	0.009
188497671	serum amyloid A2	SAA2	37.70	2	20	1.561	0.012
38016947	complement C5	C5	1.07	2	3	1.507	0.019
70906435	fibrinogen beta chain	FGB	20.98	8	13	1.499	0.020
**Down-regulated**							
6806898	alpha-synuclein	SNCA	31.25	3	3	0.692	0.010
4758328	fatty acid-binding protein, heart	FABP3	17.29	2	2	0.685	0.009
4502157	apolipoprotein C-I	APOC1	42.17	11	271	0.666	0.005
310125073	HLA A-43 alpha chain	HLA-A	17.06	1	6	0.648	0.003
32130518	apolipoprotein C-II	APOC2	56.44	7	139	0.646	0.003
4504345	hemoglobin subunit alpha	HBA1	48.59	4	21	0.639	0.002
4504349	hemoglobin subunit beta	HBD	61.90	4	60	0.481	<0.001

Coverage (%): The number of matching amino acids from peptides divided by the total number of amino acids in the sequence, expressed as a percentage; *P* values were calculated by comparing the probability of iTRAQ ratio from each group by the Maxquant software, a professional software tool for MS analysis. A *P*<0.05 indicates the protein in CHD group was significantly changed compared to the controls.

Of the 12 proteins, 7 proteins (58.3%) were consistent with well-established HDL-associated proteins including SAA2, C5, fibrinogen beta chain, apoC-I, apoC-II, hemoglobin subunit alpha, and hemoglobin subunit beta. In addition, histone H2A and HLA A-2, which respectively belong to the same functional family with histone H1 and HLA A-43 alpha chain, have been identified by shotgun proteomic analysis of HDL particles [28], [31]. A total of 3 proteins were identified for the first time as differentially expressed proteins between the two groups: ADP/ATP translocase 2, alpha-synuclein and fatty acid-binding protein.

### Functional classification of proteins associated with the HDL fraction

**Figure 2:**
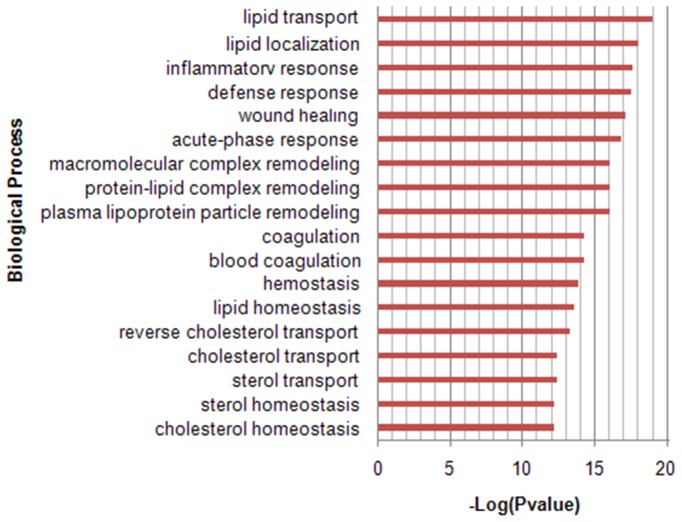
Gene ontology (GO) analysis of the 196 HDL-associated proteins for classification by biological process. According to the GO database, the main biological functions of the 196 proteins are shown with their enrichment score, represented as a *P*-value.

The biological processes and the molecular functions of all of the proteins identified in the HDL fractions were classified using a Gene Ontology (GO) classification system (Figure 2). More than 50% of the protein composition of HDL was involved in lipid and cholesterol metabolism, with lipid and cholesterol transport (18.5%), lipid localization (12.8%), lipid and cholesterol homeostasis (13.7%), and reverse cholesterol transport (5.3%) biological functions. Besides lipid and cholesterol metabolism, the proteins were associated with a broad range of biological functions such as inflammatory response, defense response, blood coagulation and acute-phase response.

The GO functional analysis indicated that the inflammatory response, acute-phase response, defense response, and platelet activation were the most significant functional processes represented by the up-regulated proteins (SAA2, C5, histone H1, fibrinogen beta chain), whereas the lipid/cholesterol transport and metabolism were the most significant functional processes represented by the down-regulated proteins (apoC-I, apoC-II, fatty acid-binding protein) (Table 3).

**Table 3:** Gene ontology (GO) category enrichments for the differentially expressed proteins in the HDL fractions.

Gene Ontology Categary	*P*-value^a^	iTRAQ expression	Gene symbol
Inflammatory response (GO:0006954)	2.16E-18	Up-regulated	SAA2, C5, FGB, H1F0
Acute-phase response (GO:0006953)	1.46E-17	Up-regulated	SAA2, FGB
Defense response (GO:0006952)	9.36E-20	Up-regulated	SAA2, C5, FGB
Platelet activation (GO:0030168)	5.75E-04	Up-regulated	SAA2, FGB
Chemotaxis (GO:0006935)	0.0452359	Up-regulated	SAA2, C5
Lipid transport (GO:0006869)	2.57E-18	Down-regulated	APOC1, FABP3, APOC2
Regulation of lipid metabolic process (GO:0019216)	7.29E-11	Down-regulated	APOC1, APOC2, SNCA
Glycerolipid metabolic process (GO:0046486)	9.92E-09	Down-regulated	APOC1, APOC2, FABP3
Regulation of fatty acid metabolic process (GO:0019217)	1.90E-06	Down-regulated	FABP3, APOC2
Regulation of hydrolase activity (GO:0051336)	1.40E-07	Down-regulated	APOC1, APOC2

a Enrichment *P*-value were calculated using a modified Fisher's exact test (EASE score).

### Confirmation of the differential levels of HDL-associated proteins in the same population of the discovery study by ELISA

Two proteins (one up-regulated (SAA) and one down-regulated (apoC-I)) were selected to confirm the changes observed in the proteomic analysis. They were chosen on the basis of their significant functions of promoting cholesterol metabolism and their involvement in an inflammatory response in the progression of CHD, according to the GO functional analysis. SAA and apoC-I were quantified in individual HDL samples of the same cohort, using ELISA kits. The protein levels were normalized to total HDL protein.

**Figure 3:**
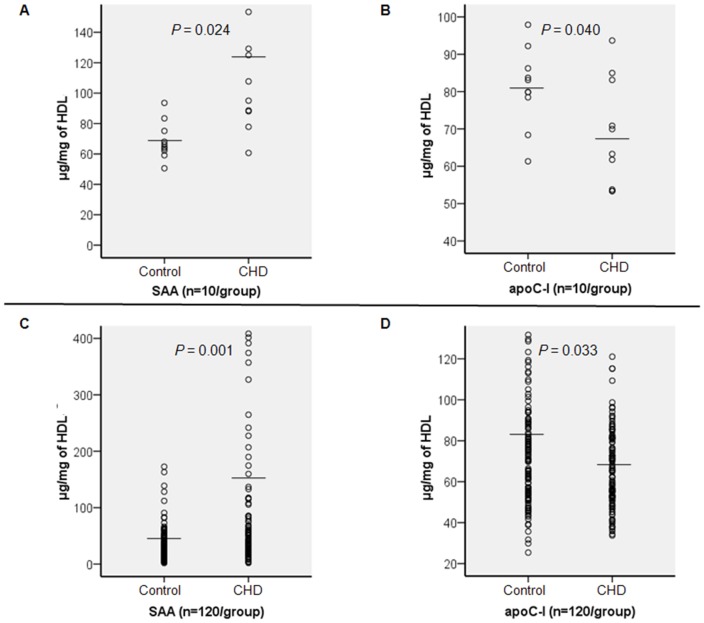
Quantified analysis of HDL-associated SAA and apoC-I. The concentration of SAA and apoC-I (µg/mg of HDL) in HDL fractions of the discovery cohort (Figure A and B) and the validation cohort (Figure C and D) were measured using ELISA kits. The mean is indicated by the horizontal line. A: The mean of SAA is 126.5±67.3 (CHD group) *vs.* 68.7±12.4 (control group) *P* = 0.024; B: The mean of apoC-I is 68.8±14.4 (CHD group) *vs.* 81.1±10.6 (control group) *P* = 0.040; C: The mean of SAA is 154.9±303.6 (CHD group) *vs.* 48.6±154.5 (control group) *P* = 0.001; D: The mean of apoC-I is 65.8±18.8 (CHD group) *vs.* 71.7±23.0 (control group) *P* = 0.033.

Expression of the proteins was consistent with the proteomic discovery study results, as HDL-associated-SAA was significantly increased in CHD individuals compared with controls (126.5±67.3 µg/mg of HDL *vs.* 68.7±12.4 µg/mg of HDL, n = 10/group, *P* = 0.024) (Figure 3A), and HDL-associated-apoC-I was significantly reduced in the CHD patients relative to the controls (68.8±14.4 µg/mg of HDL *vs.* 81.1±10.6 µg/mg of HDL, *P* = 0.040) (Figure 3B).

### Validation of differential protein abundance in an independent population by ELISA

We validated SAA and apoC-I in the HDL fraction of an independent cohort consisting of 120 CHD patients and 120 controls. The clinical characteristics of the study subjects in the validation stage are shown in Table 1. CHD patients and controls were matched for gender, age and BMI. Compared with the control group, the CHD group had significantly decreased HDL-C levels, elevated hs-CRP levels and a higher number of smokers. There were no statistical differences regarding medical treatment for antihypertensives, antidiabetics or aspirin between the two groups. ELISA data were found to be very consistent with the discovery study findings. The expression of SAA in the HDL fraction was significantly higher in CHD patients than in controls (154.9±303.6 µg/mg of HDL *vs.* 48.6±154.5 µg/mg of HDL, *P* = 0.001) (Figure 3C), and the HDL-associated apoC-I was confirmed to be significantly decreased in CHD patients compared with controls (65.8±18.8 µg/mg of HDL *vs.* 71.7±23.0 µg/mg of HDL, *P* = 0.033) (Figure 3D). In the multiple linear regression analysis, including sex, age, BMI, HDL-C, LDL-C, hs-CRP, CHD and history of smoking, hypertension and/or diabetes, HDL-associated SAA was found to be independently associated with CHD (standardized *β* = 0.2, *P* = 0.004) and hs-CRP (standardized *β* = 0.6, *P*<0.001) in the total validation population, and not associated with any other factors. HDL-associated apoC-I was negative-independently associated with CHD (standardized *β* = −0.3, *P*<0.001) and HDL-C (standardized *β* = −0.6, *P*<0.001) in the total validation population and not related to any other factors. In the CHD groups, HDL-associated SAA was significantly higher in patients with severe stenosis (Gensini >26) compared with patients with mild-to-moderate stenosis (Gensini ≤26) (*P* = 0.009). Neither gender nor medications were shown to have an impact on HDL-associated SAA and/or apoC-I in either CHD or control groups (all *P*>0.05) (Table 4).

**Table 4:** Differential expression levels of HDL-associated SAA and ApoC-I in independent CHD and control validation study population.

	CHD group			Control group		
	N*	Concentration (µg/mg of HDL)	*P*-value	N*	Concentration (µg/mg of HDL)	*P*-value
**SAA**						
Gensini ≤26^a^	60	81.9±193.5		-	-	
Gensini >26^a^	60	226.8±370.1	0.009	-	-	-
Male^b^	89	164.9±325.4		80	38.3±32.2	
Female^b^	31	126.3±233.4	NS	40	70.6±270.3	NS
No antihypertensives^c^	49	147.7±287.3		72	55.6±196.7	
With antihypertensives^c^	71	165.0±329.2	NS	48	38.1±39.0	NS
No antidiabetics^c^	104	156.6±309.0		105	52.4±177.8	
With antidiabetics^c^	16	144.3±276.0	NS	15	40.3±52.5	NS
No aspirin^c^	32	150.2±260.4		50	46.3±67.7	
With aspirin^c^	87	158.4±190.7	NS	70	50.2±162.8	NS
**ApoC-I**						
Gensini ≤26^a^	60	68.8±20.8		-	-	
Gensini >26^a^	60	63.0±16.2	NS	-	-	-
Male^b^	89	67.4±19.3		80	70.0±21.2	
Female^b^	31	61.5±16.7	NS	40	75.1±19.3	NS
No antihypertensives^c^	49	66.6±19.6		72	70.0±24.8	
With antihypertensives^c^	71	65.3±18.3	NS	48	74.4±20.0	NS
No antidiabetics^c^	104	60.0±15.3		105	69.8±23.2	
With antidiabetics^c^	16	66.8±19.2	NS	15	72.1±12.3	NS
No aspirin^c^	32	63.3±16.3		50	70.0±22.1	
With asprin^c^	87	67.5±18.2	NS	70	72.8±15.6	NS

Compared by Mann-Whitney test; NS denotes not significant between groups.

N*, sample size.

a CHD group was divided into patients with mild-to-moderate stenosis (Gensini ≤26) and patients with severe stenosis (Gensini >26), according to Gensini score (median: 26).

b CHD patients and controls were respectively divided into two groups by gender.

c CHD patients and controls were divided into patients treated with medications and patients without medications.

## Discussion

In this study, we tested the hypothesis that HDL fractions are altered during CHD, using a proteomic method, and we observed that HDL fractions transition to a pro-atherogenic profile in CHD patients. Of the differentially expressed proteins, the abundance of potential pro-atherogenic proteins was increased and the abundance of anti-atherogenic proteins was decreased. The expression levels of SAA and apoC-I were validated, confirming the reliability of our findings.

In this iTRAQ with nanoLC-MS/MS approach, we greatly improved the HDL proteome coverage by identifying 196 proteins associated with HDL, including over 90% of the previously reported proteins [4]. Twelve of the 196 proteins displayed a differential expression between the two pooled samples of CHD patients and controls. Using GO analysis, we found that the up-regulated proteins were mostly involved in inflammatory response, acute-phase response and platelet activation functional processes. One of the proteins, ADP/ATP translocase 2 has been shown to be critical in aerobic energy metabolism of myocardial cells and correlated with myocardial ischemia [32], [33]. Histones have been shown to be released during inflammation and might interact with circulating lipoproteins, and extracellular histones have been shown to play a potential pro-atherogenic role [28], [34]. Increased concentration of C5 and its degradation products have been suggested to be strongly associated with plaque rupture and onset of acute cardiovascular events [35]. Another increased protein, fibrinogen, has been suggested as a biomarker of inflammation for prediction of cardiovascular events [36].

Among the up-regulated proteins, HDL-associated SAA was confirmed to be higher in CHD patients compared to controls. To our knowledge, this is the first study to quantitatively detect a significant elevation of SAA in HDL fractions of CHD patients, i.e. not only in plasma. Moreover, the HDL-associated SAA was found to be independently associated with plasma hs-CRP, an inflammatory marker, and thus linked the inflammatory conditions to HDL protein composition. SAA is an acute-phase protein with numerous pro-inflammatory actions and its concentration increases up to 1000 times in the blood during inflammatory responses [37], [38]. Once in circulation, SAA rapidly and primarily associates with HDL, which renders HDL pro-atherogenic with a loss or reduction of many of its protective functions [10], [26], [39]. An increased level of SAA in plasma has been associated with cardiovascular disease and it might play a contributory role in atherosclerosis development [24], [40]–[43]. SAA was superior to hs-CRP in association with CHD and can be independently predictive of adverse cardiovascular outcomes[44]. To our knowledge, this is the first study showing that HDL-associated SAA is related to the degree of coronary artery stenosis. Therefore, these data suggest that the enrichment of SAA in HDL during CHD might reduce the anti-atherogenic properties of HDL reduce the anti-atherogenic properties of HDL [18].

Different from our observations, a small sample study (n = 10/group) by Alwali *et al.* reported that HDL from acute myocardial infarction (AMI) patients expressed an inflammatory profile compared to controls, but not in the stable CHD patients [45]. Nevertheless, in that study, all of the stable CHD patients and 10% of controls, but not the AMI patients, were receiving regular statins therapy, which has anti-inflammatory effects and has been demonstrated to promote the formation of a more favorable HDL fraction profile and increase HDL-C levels [16], [46], [47]. In our study, we excluded subjects receiving anti-inflammatory medications and/or lower lipid therapy, such as statins. In addition, we did not enroll AMI patients because AMI is complicated by numerous acute proteins and inflammatory factors and that might be considered as a limitation of this study.

For the down-regulated proteins, lipid/cholesterol transport and metabolism were the most involved functional processes. Firstly, heart-type fatty acid-binding protein is believed to play a role in the intracellular transport of long-chain fatty acids that could decrease the risk of cardiovascular disease via the favorable effects on anti-inflammation [48], [49]. Secondly, apoC-I is a secreted plasma protein and binds with HDL, LDL and VLDL in the circulation. ApoC-I has been demonstrated to aid in the synthesis and stabilization of mature HDL particles by activating lecithin cholesterol acyl transferase and inhibiting cholesterol ester transfer protein and hepatic lipase (CETP) [50], [51]. In this study, for the first time, we confirmed a significant decrease in HDL-associated apoC-I in CHD patients, which might contribute to hampering the maturation of the HDL and affect its protective function. While, it has been reported that inhibition of plasma CETP by apoC-I is blunted in CHD patients with dyslipidemia, this is probably due to increasing amounts of VLDL-bound apoC-I, which is inactive as a CETP inhibitor[52]. It might be interesting to test ApoC-I in other lipoproteins as well as the relative enzymes in future studies. In addition, apoC-II deficiency was demonstrated to affect the maturation of HDL [53], and low apoC-II levels have been shown to be associated with cardiovascular mortality [54], [55]. Two cases of ApoC-II deficiency syndrome were reported with elevation of plasma triglycerides, and it was corrected by the apolipoprotein C-II administration [56]. However, further studies are necessary to investigate the impact of the decreased apoC-I and II on HDL function in CHD patients.

We also identified down-regulated hemoglobin in CHD patients. HDL-associated hemoglobin is thought to contribute to the pro-inflammatory nature of HDL in CHD patients, and HDL from CHD patients has been demonstrated to contain more hemoglobin [57], [58]. In contrast, we observed a significant decrease in HDL-associated hemoglobin in CHD patients relative to controls (Table 2), which may be related to the significant decrease in total hemoglobin levels in the CHD group compared to controls (137.3±13.3 g/dl *vs.* 144.6±17.0 g/dl, *P*<0.05) [45]. Less likely to be associated with HDL are alpha-synuclein, a neuronal protein [59], and HLA-A43, a human leukocyte antigen. However, HLA-A2, belonging to the same functional family of human leukocyte antigens as HLA-A43, was reported to be detected in HDL fractions [31]. Further studies are needed to elucidate the role of HLA-As in HDL function and in CHD patients.

One potential limitation is the use of ultracentrifugation for HDL isolation, which might strip off proteins during the procedure, possibly resulting in an under-estimation of the HDL composition. However, ultracentrifugation has been well established as a method of HDL isolation and is widely used to date [4], [27]. Another limitation in our study is that females were excluded from the proteomic analysis to eliminate potential confounding bias of menopausal status. However, we did include females in the validation stage, and no significant differences were detected in HDL-associated apoC-I and SAA between genders in either group.

In conclusion, the HDL proteome displays a pro-atherogenic profile that might compromise the protective effects of HDL in CHD patients. Enrichment of SAA has a potential relevance to the pro-inflammatory properties of HDL. Recent studies have stressed that the simple concentration of HDL-C may not directly reflect HDL function [60], [61]. Assessment of the direct contributions of HDL particles to cardiovascular prediction and prevention requires investigation of HDL-related biomarkers that are more tightly associated with critical anti-atherosclerotic effects of HDL than HDL-C [8]. Therefore, additional proteomic measurement of HDL particles could aid in detecting these special HDL-associated proteins and, in turn, in evaluating the risk of cardiovascular events and identifying a more appropriate therapeutic target than HDL-C levels.

## Supporting Information

Table S1 **The two density cutoff values of ultracentrifugation technique for HDL isolation.**
(XLSX)

Table S2 **The purity of 20 random HDL samples.**
(XLS)

Table S3 **Protein summary report identified in HDL samples by iTRAQ.**
(XLSX)
